# Genetic Diversity and Structure of *Coix lacryma-jobi* L. from Its World Secondary Diversity Center, Southwest China

**DOI:** 10.1155/2019/9815697

**Published:** 2019-01-21

**Authors:** Yu-Hua Fu, Chenglong Yang, Qiuyi Meng, Fanzhi Liu, Gang Shen, Mingqiang Zhou, Maohong Ao

**Affiliations:** Guizhou Institute of Subtropical Crops, Guizhou Academy of Agricultural Sciences, 1 Fenglindong Road, Xingyi 562400, China

## Abstract

*Coix lacryma-jobi* L. is an important minor cereal with a high nutritional and medicinal value in Asian countries. The hilly region of South China is the secondary center of diversity of *Coix lacryma-jobi* L. In the present study, we took a sample of 139 *Coix lacryma-jobi* L. genotypes from four geographical regions in Southwest China and analyzed the genetic diversity and population structure using AFLP markers. Six primer combinations detected a total of 743 (89.52%) polymorphic loci. The percentage of polymorphic bands within the four geographical populations ranged from 56.02% (Guangxi) to 86.75% (Guizhou). The overall genetic diversity of 139 *Coix lacryma-jobi* L. was relatively low (*h* ranged from 0.1854 to 0.2564). The neighbor-joining method grouped all *Coix lacryma-jobi* L. genotypes into two clusters with no geographical affinity observed among genotypes within the same group. The Fst indicated the two clusters existed great genetic differentiation. AMOVA analysis showed the molecular variation within populations was much higher than that among populations of geographical regions and subpopulations derived from STRUCTURE. Human activities and the natural outcrossing system of *Coix lacryma-jobi* L. may have a great influence on its distribution, genetic diversity, and population structure. Our study provides useful information for local breeding programs of *Coix lacryma-jobi* L. as well.

## 1. Introduction

*Coix lacryma-jobi* L., also commonly known as adlay or Job's tear, is a tall, robust, and strongly tillering grass belonging to the family Poaceae. It is native to Asia and now has been naturalized throughout the tropics and subtropics about 22°N and S [1, 2]. *Coix lacryma-jobi* L. has higher protein content than other cereals that makes it a good source of nutrition for humans and animals [1, 3, 4]. Before maize became popular in South Asia, this plant was an important minor cereal and became a minor food with economic utilities and fodder crop in north India [5]. People in China and Korea use the roots and leaves, especially the kernels of *Coix lacryma-jobi* L., as a remedy in traditional medicine [1]. Recent pharmacological studies have shown the medicinal properties of *Coix lacryma-jobi* L., such as antioxidant activity [6, 7], anti-inflammatory activity [8, 9], antidiabetic activity [10], antiallergic activity [11], antiobesity activity [12, 13], and anticancer activity [14–16].

*Coix lacryma-jobi* L. is a cultivated species in the genus *Coix* which has 9 to 11 species. Southwest China is the secondary center of diversity of *Coix lacryma-jobi* L., and Guangxi (GX) might be one of its origin areas [1, 17, 18]. It is documented that *Coix lacryma-jobi* seed was found at the Hemudu site, indicating that *Coix lacryma-jobi* has been cultivated in China for more than 6000 years [19]. The current planting area of *Coix lacryma-jobi* L. in China is estimated around 73,000 ha with a grain yield of 0.22 million tons [20]. Guizhou (GZ), especially southwest Guizhou, has become the largest producer and distribution center for *Coix lacryma-jobi* L. both in China and in Southeast Asia [20, 21].

Diversity analysis of a particular species or population is one of the key aspects of research for germplasm evaluation and crop genetic improvement, conducive to protection and effective utilization of germplasm resources. DNA molecular markers are a powerful tool for diversity analysis by detecting the genetic variations between individuals. Li et al. first performed genetic evaluation on *Coix* germplasm using RAPD (random amplified polymorphic DNA) [22]. Ma et al. assessed the genetic relationship among 79 *Coix lacryma-jobi* accessions and found accessions from GX, China possessed greater genetic diversity than the accessions originating from Korea [23]. Guo et al. evaluated 22 *Coix lacryma-jobi* L. accessions using SSR (simple sequence repeats) developed from maize and rice [24]. Wang et al. studied the genetic diversity of 25 *Coix lacryma-jobi* L. using SRAP (sequence-related amplified polymorphism) markers [25]. Compared with other dominant markers, AFLP (amplified fragment length polymorphism) can produce abundant and stable genetic markers, capable of discriminating closely related accessions [26, 27]. It has been successfully applied in molecular characterization and genetic structure studies in various plants such as soybean [28], linseed [29], and wild banana [30].

In this study, we aim to assess the genetic diversity and population structure of 139 genotypes of *Coix lacryma-jobi* L. from China using AFLP markers. To our knowledge, this is the first attempt to characterize the genetic variability amongst *Coix lacryma-jobi* L. genotypes from four regions of southwest China using molecular markers. The results obtained from this study will provide useful information for germplasm utilization and other related studies in the future.

## 2. Materials and Methods

### 2.1. Plant Materials

**Figure 1:**
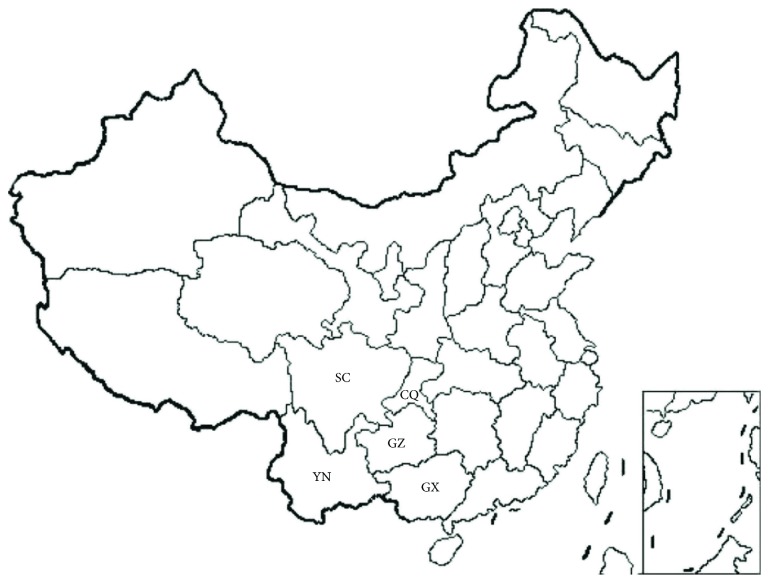
Geographical map showing the distribution of sampling regions.

139 genotypes of *Coix lacryma-jobi* L. collected from four regions of southwest China (as shown in Table 1 and Figure 1) were grown in a germplasm nursery at Guizhou Institute of Subtropical Crops. The morphological characteristics of these germplasm were measured, including plant height, tiller number, nod on main stem, branch number, leaf length, leaf width, and hundred-grain weight (Table S1). Young leaves from each genotype were collected and stored at −20°C for total DNA isolation. DNA extraction was performed using the CTAB method as described by Doyle and Doyle [31]. DNA quality and concentration were measured by running 0.8% agarose gel, employing diluted uncut *λ* DNA as standard. DNA final concentration was adjusted to 50 ng/*μ*L in TE and stored at −20°C for AFLP analysis.

### 2.2. AFLP Analysis

AFLP analysis was carried out using the protocol by Vos et al. [32] with some modifications. Adaptor and primer sequences used in AFLP reaction are shown in Table 2. Genomic DNA (approximately 250 ng) was digested with 4 U *Eco*RI and 4 U *Mse*I (Fenmentas, USA) for 3 h at 37°C and then at 65°C for 3 h. Restricted DNA fragments were ligated to the *Eco*RI adaptor (50 *μ*M) and *Mse*I adaptor (50 *μ*M) using T4 ligase (5 U/*μ*L, Fermentas, USA) at 16°C overnight. The ligation product was diluted five times with ddH_2_O and then used as a template for preamplification. 2 *μ*L of adaptor-ligated DNA was preamplified with primers E_00_ and M_00_ using the thermocycle carried out as follows: 94°C for 3 min, followed by 26 cycles of 94°C for 45 s, 50°C for 45 s, 72°C for 1 min, followed by 72°C for 10 min. The selective amplification was subsequently conducted using different combinations of FAM-labeled *Eco*RI primers and unlabeled *Mse*I primers with three selective nucleotides. The preamplified product (20-fold diluted) was used as the template for the selective amplification in a total volume of 20 *μ*L. The selective amplification was carried out using touchdown PCR programs as follows: 95°C for 5 min, 12 cycles of 95°C for 35 s, 65°C (−0.7°C per cycle) for 35 s, 72°C for 1 min, 23 cycles of 94°C for 30 s, 56°C for 30 s, 72°C for 1 min, and final extension at 72°C for 10 min. 1 *μ*L of the diluted (10-fold) labeled PCR product combined with 15 *μ*L solution of Hi-Di formamide and internal size standard LIZ-500 (volume ratio was 100 : 1) was analyzed via capillary electrophoresis on a DNA analyzer ABI3730XL (Applied Biosystems). The fragment analysis was carried out by the GeneMarker software.

### 2.3. Data Analysis

The AFLP fragments in the range of 35–500 bp were obtained and scored as present (1) or absent (0) to generate a binary data matrix. The binary matrix was used to analyze the polymorphic features of each primer combination such as percentage polymorphism, polymorphism information content (PIC), Nei's genetic diversity index (*h*), and Shannon's index (*I*) using POPGENE32 [33]. The effective multiplex ratio (EMR) and marker index (MI) for each marker were calculated according to the method by Powell et al. [34]. A dendrogram was constructed using software Mega 5.0 on the basis of a neighbor-joining method [35]. The genetic population structure of 139 genotypes was analyzed using software STRUCTRE 2.3.4 [36] and Structure Harvester (http://taylor0.biology.ucla.edu/structureHarvester/). The optimum number of populations (*K*) was set in the range of 2 to 15. A burn-in period length of 10,000 followed by 100,000 MCMC (Markov chain Monte Carlo) repetitions was run ten times for each value of *K* number. The optimum *K* value was determined according to the method by Evanno et al. [36]. Arlequin 3.1.0.2 [37] was used to calculate the Fst (fixation index) and 1000 Bootstrap replication was applied for testing the confidence level of the Fst. Analysis of molecular variance (AMOVA) among 139 genotypes was assessed using GENALEX 6.5 [38] on the basis of both geographical regions and different groups derived from population structure analysis.

## 3. Results

### 3.1. AFLP Polymorphism

Six primer combinations generated a total of 830 AFLP bands from 139 *Coix lacryma-jobi* L. genotypes of four geographical populations, of which 743 bands (89.52%) were polymorphic. The details of marker features for each primer combination are given in Table 3. On average, each primer combination generated 138.3 bands, ranging from 106 to 171 bands. The number of polymorphic bands per primer combination varied from 89 to 161 with an average of 123.8. The primer combination E48M85 and E45M55 produced the highest (94.15%) and lowest (82.04%) polymorphic percentages, respectively. PIC values for the six primer combinations ranged from 0.165 to 0.242 with an average of 0.202. The marker index varied from 12.55 to 33.05 with an average of 22.68 per primer combination. The highest value (33.05) was observed for the primer combination E48M85, and the lowest value (12.55) was recorded for the primer combination E47M58. Together, the primer combination E48M85 was considered the most informative among the six AFLP primer combinations used in this study.

### 3.2. Genetic Diversity and Cluster Analysis

**Figure 2:**
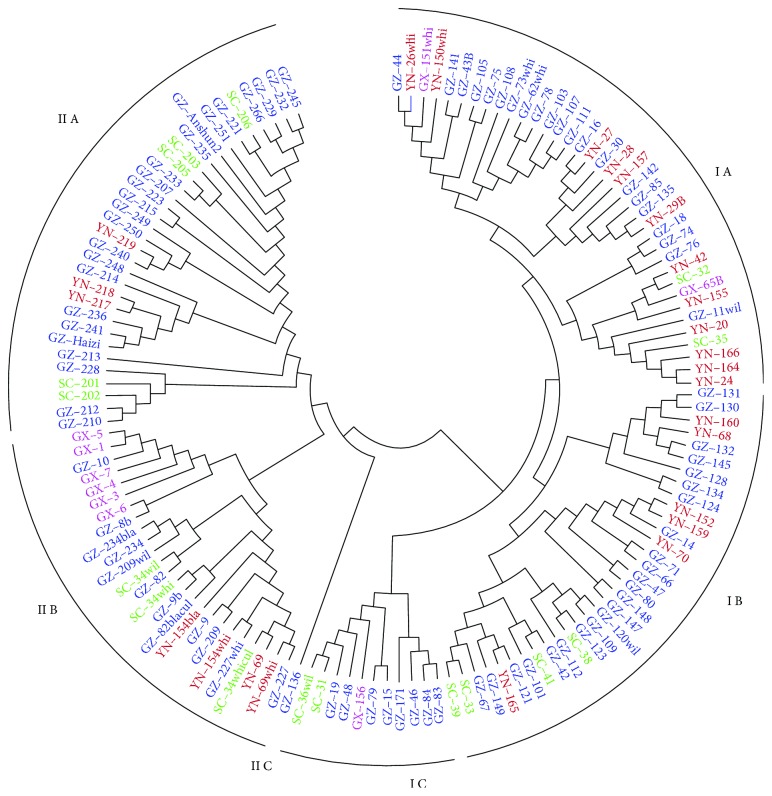
Genetic relationship among 139 *Coix lacryma-jobi* L. genotypes based on neighbor-joining clustering.

AFLP genotyping data obtained for six primer combinations were analyzed using the neighbor-joining method. A dendrogram was constructed grouping 139 *Coix lacryma-jobi* L. genotypes into two major clusters, namely, I and II (Figure 2). Cluster I was found to be the larger cluster with 81 genotypes and could be further subdivided into subclusters IA, IB, and IC with 37, 33, and 11 genotypes, respectively. Subcluster IA showed mixed genotypes from all regions including 21 from GZ, 12 from YN, 2 from GX, and 2 from SC. Genotypes from GX were completely absent in subcluster IB, and genotypes from YN were absent in subcluster IC. Cluster II consisted of 58 genotypes. It could be further subdivided into clusters IIA (32 genotypes), IIB (25 genotypes), and IIC (1 genotype), respectively. Subcluster IIA had genotypes from GZ (24), SC (5), and YN (3). In subcluster IIB, a majority of genotypes from GX was closely grouped along with 12 GZ, 4 YN, and 3 SC. Only one genotype from GZ was in subcluster IIC.

At the geographical level, results of genetic analysis of the four *Coix lacryma-jobi* L. populations are shown in Table 4 for Nei's gene diversity (*h*), Shannon's information index (*I*), and percentage polymorphism (PP). Nei's gene diversity values ranged from a minimum of 0.1854 to a maximum of 0.2564, Shannon's information index values varied from 0.2809 to 0.3906, and the highest percentage polymorphism were observed for the population of GZ (86.75%) followed by YN (77.59%), SC (70.60%), and GX (56.02%). The values of Nei's gene diversity, Shannon's information index, and percentage of polymorphic bands were all highest in GZ and lowest in GX.

**Figure 3:**
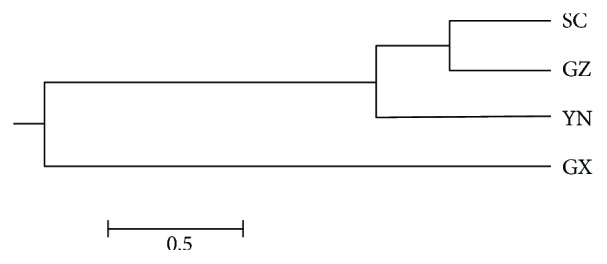
Dendrogram-based Nei's (1978) genetic distance using the UPGMA (unweighted pair group method analysis) method.

The pair-wise genetic distance values were also calculated for all four populations (Table 5). Nei's genetic distance varied from 0.0075 to 0.0469 while the genetic identity between populations ranged from 0.9542 to 0.9926. Population of SC and GZ had the lowest genetic distance of 0.0075, and SC and GX had the highest genetic distance of 0.0469. The UPGMA dendrogram grouped the four populations into three major clusters (Figure 3). One is composed of SC and GZ, and the other two included YN and GX.

### 3.3. Population Structure

**Figure 4:**
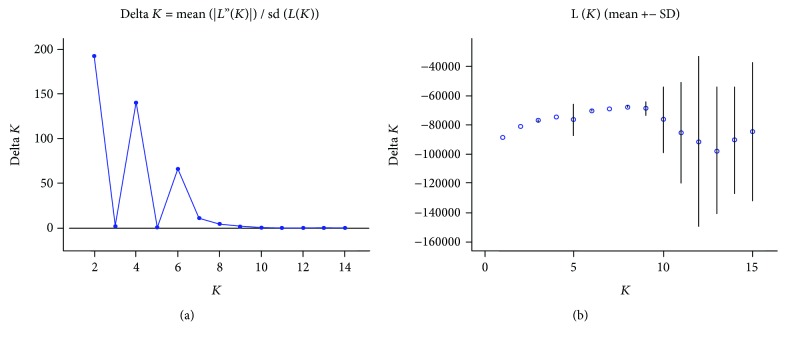
Values of delta *K* (a) and *L*(*K*) (b).

**Figure 5:**
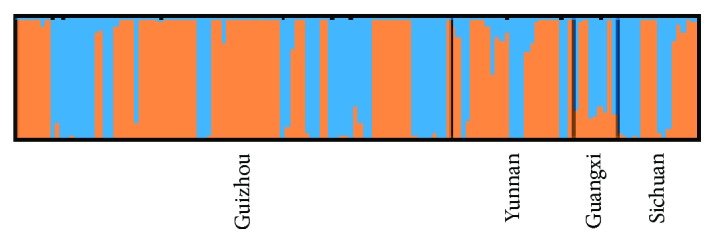
STRUCTURE analysis identified two subpopulations among 139 *Coix lacryma-jobi* L. genotypes.

The number of genetically distinct populations was analyzed using STRUCTURE program. The highest value of delta *K* based on the Bayesian clustering method was scored when *K* equaled 2 (Figure 4). Therefore, two subpopulations among 139 *Coix lacryma-jobi* L. genotypes were determined with each subpopulation including 82 genotypes and 57 genotypes (Figure 5). The bigger subpopulation contained genotypes from GZ, SC, YN, and GX with 53, 8, 18, and 3, respectively. The smaller subpopulation consisted of a majority of 36 genotypes from GZ followed by 8 from SC, 7 from YN, and 6 from GX.

AMOVA analysis was performed on both geographical regions and genetic subpopulations as shown in Table 6. At the geographical region level, the significant variation (97%) was observed among the individuals within geographical regions while only 3% of the total molecular variation was found among regions. The two subpopulations derived from STRUCTURE showed 17% and 83% of total variance among and within the subpopulations, respectively.

## 4. Discussion

This is the first report of genetic characterization of *Coix lacryma-jobi* L. from four regions in southwest China. For in-depth genetic diversity assessment, 139 *Coix lacryma-jobi* L. genotypes from four regions, namely, GZ, GX, SC, and YN, were analyzed using AFLP technology. The present study determined the feasibility of the use of AFLP markers in establishing a genetic relationship among the *Coix lacryma-jobi* L. growing under karst climate. Previous studies have shown that AFLP is a powerful tool for characterization and evaluation genetic variation among various crops including *Coix*'s closest genera *Zea*, *Tripsacum*, and *Sorghum*. Garcia et al. [39] reported that AFLP markers were the best choice for the evaluation of diversity and the assessment of the genetic relationships between tropical maize inbred lines with high accuracy. Our study showed that AFLP analysis generated a large number of polymorphic bands in *Coix lacryma-jobi* L. revealing relatively high genetic diversity (89.52% polymorphism) in comparison with previous studies in other crops, e.g., 72% polymorphism in maize [40] and 85% polymorphism in sorghum [41]. This indicates AFLP markers have high discriminating power and are suitable for molecular fingerprinting of *Coix lacryma-jobi* L. Three parameters, PIC, EMR, and MI, were also calculated to evaluate the AFLP system applied in *Coix lacryma-jobi* L. where MI is considered a measure of the overall efficiency of a marker system [34]. Markers with a higher value of MI and EMR are more suitable for distinguishing and determining genetic variability. Therefore, marker E48M85 is the most informative AFLP primer combination in the present study followed by E38M51. These markers are recommended for assessing the genetic diversity of *Coix lacryma-jobi* L. genotypes growing under other climates.

Based on our genotyping data, the overall genetic diversity of *Coix lacryma-jobi* L. from GZ, GX, YN, and SC is relatively low, *h* ranging from 0.1854 to 0.2564 and *I* ranging from 0.2809 to 0.3906, consistent with Xi et al.'s study that *Coix lacryma-jobi* L. had low genetic diversity at both the species level and the accession level [42]. Moreover, several studies on genetic variation of *Coix lacryma-jobi* L. also showed relatively high genetic similarity among *Coix lacryma-jobi* L. genotypes, such as 0.30~0.92 with an average of 0.78 in Guo et al.'s study [24], 0.48~0.82 in Wang et al.'s study [25], and 0.30~0.81 in Li et al.'s study [22]. The dendrogram result of 139 *Coix lacryma-jobi* L. was not region specific. In other words, genotypes collected from the same geographical region differed genetically. Genotypes from GZ, SC, and YN were scattered all over the dendrogram revealing higher levels of polymorphism while genotypes from GX, 6 out of 9 were grouped closely in one subcluster. This was in agreement with the level of polymorphism in *Coix lacryma-jobi* L. from the four different regions. GX had the lowest polymorphism (56.02%) while GZ had the highest polymorphism (86.75%). However, it was discordant with our expectation that *Coix lacryma-jobi* L. from GX should be more genetically diverse since GX was considered to be one of origin areas for *Coix lacryma-jobi* L. [17, 18]. One reason for this could be the sample sources and population size. Only 9 *Coix lacryma-jobi* L. from GX were used for this study, and they were all from places adjacent to GZ. Therefore, the 9 genotypes did not represent the extent of genetic variations of *Coix lacryma-jobi* L. in GX. And this is the case with YN as well. Thus, a further study with a larger collection of *Coix lacryma-jobi* L. from different districts in GX, YN, and SC should be carried out. Another reason might be due to anthropological activities such as people who brought the seed to a new place to grow when they migrated, that would cause the fact that the true origin of the genotypes was not where they were collected. Economic development and government policy also have a great influence on the local distribution of *Coix lacryma-jobi* L. It is reported the habitat of wild *Coix lacryma-jobi* L. and *Coix aquatica* Roxb. in China has been damaged increasingly, compared with that in 80s of the last century, 70% wild *Coix* habitat in GX was destroyed [19], while GZ has become the area with the largest planting areas and the highest yield production in China as well as the largest distributing center in both China and Southeast Asia [20, 21]. Therefore, *Coix lacryma-jobi* L. from outside GZ or even China could likely be introduced into GZ, stored and planted by local farmers, resulting in increasing the *Coix lacryma-jobi* L. diversity in GZ. This may partially explain the reason that GZ had higher polymorphism than GX. Moreover, the result of the clustering of the four populations showed three groups were formed: GZ and SC populations presented in one group, and YN and GX populations were the other two groups. This may indicate the four *Coix lacryma-jobi* L. populations were originally from some areas in GX and then differentiated highly due to human activities like moving across regions or selective breeding over the course of time.

Population structure analysis by STRUCTURE was generally in line with clustering based on genetic distance as they both divided 139 *Coix lacryma-jobi* L. into two groups, but one genotype GZ-136 was not consistent. GZ-136 was in the bigger subpopulation by STRUCTURE while it was in subcluster IIC of the smaller group in the dendrogram. Note that GZ-136 was the only genotype in subcluster IIC indicating it, to a certain degree, was not genetically close to the other genotypes in the smaller cluster. The Fst among the two clusters was 0.168 indicating the two clusters existed great genetic differentiation according to Wright [43]. Moreover, higher genetic variation was found within populations, both geographically and genetically. Besides the cause of human activities during the long-term domestication of *Coix lacryma-jobi* L., it may be due to the breeding system of *Coix lacryma-jobi* L. Mello et al. [2] investigated the breeding systems in six *Coix lacryma-jobi* populations, the rate of natural outcrossing of *C. lacryma-jobi* ranged from 35.9% to 72.8%. Thus, the mixed breeding system may account for it having higher genetic variation within populations but not among them [44].

## 5. Conclusions

139 *Coix lacryma-jobi* L. from four regions in southwest China, the world secondary diversity center, were analyzed genetic diversity and population structure using AFLP markers. Six primer combinations identified a total of 743 polymorphic bands. The neighbor-joining method grouped all *Coix lacryma-jobi* L. genotypes into two clusters with no geographical affinity observed among genotypes within the same group. The Fst indicated the two clusters existed great genetic differentiation, but the overall genetic diversity of 139 *Coix lacryma-jobi* L. was relatively low; *h* ranged from 0.1854 (GX) to 0.2564 (GZ). *Coix lacryma-jobi* L. from GZ showed a higher level of genetic diversity compared to genotypes from other three regions. That may be due to the sample size difference among the four regions and human activities that affected the habitat and distribution of *Coix lacryma-jobi* L. AMOVA analysis showed that the molecular variation within populations was much higher than that among populations of geographical regions and subpopulations, which may be explained by the natural outcrossing system of *Coix lacryma-jobi* L.

## Figures and Tables

**Table 1:** General information of 139 *Coix lacryma-jobi* L. collected from four regions of Southwest China.

Region	Abbreviation	Sample size
Guizhou	GZ	89
Guangxi	GX	9
Yunnan	YN	25
Sichuan (including CQ)	SC	16
Total		139

**Table 2:** The sequences of adapters and primers used for AFLP analysis.

Name	Sequence (5′-3′)
Adapter	
*Eco*R I adapter	CTCGTAGACTGCGTACC
	AATTGGTACGCAGTCTAC
*Mse*I adapter	GACGATGAGTCCTGAG
	TACTCAGGACTCAT
Pre-selective primers	
E00	GACTGCGTACCAATTC
M00	GATGAGTCCTGAGTAA
Selective Primers	
E38	GACTGCGTACCAATTCACT
E39	GACTGCGTACCAATTCAGA
E45	GACTGCGTACCAATTCATG
E47	GACTGCGTACCAATTCCAA
E48	GACTGCGTACCAATTCCAC
E49	GACTGCGTACCAATTCCAG
M51	GATGAGTCCTGAGTAACCA
M54	GATGAGTCCTGAGTAACCT
M55	GATGAGTCCTGAGTAACGA
M58	GATGAGTCCTGAGTAACGT
M85	GATGAGTCCTGAGTAATCG
M86	GATGAGTCCTGAGTAATCT

**Table 3:** Details of marker features of six AFLP primer combinations.

Primer combination	Total number of bands	Polymorphic bands	Percentage polymorphism (%)	PIC^a^	EMR^b^	MI^c^
E38M51	144	134	93.06	0.211	124.69	26.31
E39M54	120	110	91.67	0.206	100.83	20.77
E45M55	167	137	82.04	0.165	112.39	18.54
E47M58	106	89	83.96	0.168	74.73	12.55
E48M85	171	161	94.15	0.218	151.58	33.05
E49M86	122	112	91.80	0.242	102.82	24.88
Total	830	743				
Mean	138.3	123.8	89.52	0.202	111.17	22.68

^a^Polymorphism information content. ^b^Effective multiplex ration. ^c^Marker index.

**Table 4:** Summary of genetic variation statistics for *Coix lacryma-jobi* L. from four geographical regions.

Geographical region	*h* ^1^	*I* ^2^	PP (%)^3^
SC	0.2400	0.3602	70.60
YN	0.2415	0.3670	77.59
GX	0.1854	0.2809	56.02
GZ	0.2564	0.3906	86.75

^1^ *h* = Nei's (1973) gene diversity. ^2^ *I* = Shannon's information index [Lewontin (1972)]. ^3^Percentage of polymorphic bands.

**Table 5:** Nei's genetic identity (above diagonal) and genetic distance (below diagonal) among four populations.

Geographic region	SC	YN	GX	GZ
SC	^∗∗∗∗^	0.9820	0.9542	0.9926
YN	0.0181	^∗∗∗∗^	0.9717	0.9922
GX	0.0469	0.0287	^∗∗∗∗^	0.9633
GZ	0.0075	0.0078	0.0374	^∗∗∗∗^

**Table 6:** Summary of AMOVA results of 139 *Coix lacryma-jobi* L. genotypes among and within populations in geographical regions and subpopulations derived from STRUCTURE.

Source	df	SS	MS	Est. Var.	% Var.	*P* value^∗^
Geographical locations						
Among Pops	3	566.727	188.909	3.466	3%	0.001
Within Pops	135	13789.719	102.146	102.146	97%	
Total	138	14356.446		105.612	100%	
Subpopulations derived from STRUCTURE (*K* = 2)						
Among Pops	1	1369.290	1369.290	19.057	17%	0.001
Within Pops	137	12987.156	94.797	94.797	83%	
Total	138	14356.446		113.854	100%	

df = degrees of freedom; SS = the sums of squares; MS = the mean sums of squares; Est. Var. = the estimated variance; % Var. **=** percentage of variation. ^∗^With 999 data permutations.

## Data Availability

The AFLP data used to support the findings of this study have been deposited in the MTS repository (Supplementary Materials file).

## Supplementary Materials

Supplementary Materials Supplementary Table S1: morphological characteristics of 139 *Coix lacryma-jobi* L. genotypes.
